# Frequency-dependent modulation of foveal contrast sensitivity by fine-scale exogenously triggered attention

**DOI:** 10.7554/eLife.108788

**Published:** 2026-06-29

**Authors:** Yue Guzhang, T Florian Jaeger, Martina Poletti

**Affiliations:** 1 Department of Brain and Cognitive Sciences, University of Rochester Rochester United States; 2 Goergen Institute for Data Science and Artificial Intelligence, University of Rochester Rochester United States; 3 Center for Visual Science, University of Rochester Rochester United States; 4 Department of Neurosciences, University of Rochester Rochester United States; Wake Forest University School of Medicine United States; Peking University China

**Keywords:** visual attention, contrast sensitivity, eye movements, Human

## Abstract

Exogenous attention is a rapid, involuntary mechanism that automatically reallocates processing resources toward salient stimuli. It enhances visual sensitivity in the vicinity of the salient stimulus, both in extrafoveal regions and within the high-acuity foveola. While the spatial frequencies (SFs) modulated by exogenous attention in extrafoveal vision are well characterized, it remains unknown how this mechanism operates within the foveola, which can resolve SFs up to 30 cycles per degree (CPD). Here, we examined which SFs were enhanced by fine-grained deployments of exogenous attention within this highest-acuity region of the visual field. Using high-precision eye-tracking to precisely localize gaze during attentional allocation, we found that exogenous attention at the foveal scale selectively enhances contrast sensitivity for low- to mid-range SFs (4–8 CPD), with no significant benefits for higher SFs (12–20 CPD). In contrast, attention-related benefits on asymptotic performance at the highest contrast were observed across a wide range of SFs. These results indicate that, despite the high-resolution capacity of the foveola, exogenous attention remains an inflexible mechanism that, even at this scale, selectively enhances contrast gain for lower SFs—mirroring its behavior in extrafoveal vision.

## Introduction

Visual spatial attention is a fundamental mechanism that enables both humans (Carrasco, 2011) and animals (Saban et al., 2017; Hahner and Nieder, 2025) to selectively process information from their environment. Often, shifts in spatial attention are accompanied by eye movements to focus on a specific location, a process known as overt spatial attention (Kaspar, 2013). However, covert spatial attention, the ability to shift attention independently of eye movements, is equally crucial in daily life. This ability enables us to monitor locations beyond our line of sight, such as when driving and keeping track of peripheral surroundings.

Covert spatial attention is typically categorized into two types: endogenous and exogenous attention. Endogenous attention refers to the voluntary allocation of processing resources to a specific location. While this shift occurs relatively slowly, taking approximately 200–300 ms to reach the target region, it can be sustained for an extended duration (Theeuwes, 1994; Findlay, 2003; Chica and Lupiáñez, 2009; Carrasco, 2011; Chica et al., 2013; Dugué et al., 2020). In contrast, exogenous attention is driven by salient stimuli that automatically capture attention (Theeuwes, 1994; Findlay, 2003; Chica and Lupiáñez, 2009; Carrasco, 2011; Chica et al., 2013; Dugué et al., 2020). This shift is rapid but transient, often followed by a phenomenon known as inhibition of return, moving attention away from the initially attended location (Klein, 2000; Chica and Lupiáñez, 2009). Compared to endogenous attention, exogenous attention is more automatic and less flexible (Corbetta and Shulman, 2002; Knudsen, 2007; Carrasco, 2011).

Until recently, research on the effects of covert attention on visual perception has focused primarily on extrafoveal vision. A vast body of literature has demonstrated that covert attention enhances visual contrast sensitivity (CS) (Carrasco et al., 2000; Martínez-Trujillo and Treue, 2002; Reynolds and Chelazzi, 2004; Pestilli and Carrasco, 2005; Li et al., 2008; Foster et al., 2021) and increases spatial resolution (Yeshurun and Carrasco, 1998; Carrasco et al., 2002; Carrasco et al., 2006; Jigo and Carrasco, 2018) at selectively cued locations in the extrafovea. In contrast, attention within the high-acuity 1° foveola has often been considered uniform and distributed evenly throughout this small region. Therefore, the effects of attention in the fovea are traditionally studied using large stimuli encompassing one or more degrees of visual angle (Miniussi et al., 2002; Jigo and Carrasco, 2020; Papaioannou and Luck, 2020). However, recent findings showed that even within the 1° foveola, both endogenous (Poletti et al., 2017) and exogenous (Guzhang et al., 2021) attention can be covertly allocated in a highly spatially selective manner. For both types of covert attention, observers were better able to discriminate the orientation of fine details at an attended location—cued endogenously or exogenously—compared to nearby uncued locations just 0.26° away. Although these results highlighted the strikingly fine grain of attentional control, they also raised new questions. In particular, it remains unknown which spatial frequencies (SFs) benefit from fine-grained attentional shifts within the foveola. While Guzhang et al., 2021 demonstrated visual enhancement from exogenous attention at the foveal scale, the orientation discrimination task used in the study was relatively coarse, requiring observers to determine whether a stimulus was tilted ±45°. Despite the small size of the stimulus, such a task does not require high SFs (e.g., >10 cycles per degree [CPD]); in fact, frequencies around 4–8 CPD should be sufficient to perform it effectively. Therefore, the perceptual enhancement observed in Guzhang et al., 2021 could be due to an enhancement of only lower or only higher SFs, or perhaps a broad range of SFs. The overall improvement in orientation discrimination of fine spatial stimuli is compatible with any of these scenarios.

**Figure 1.**
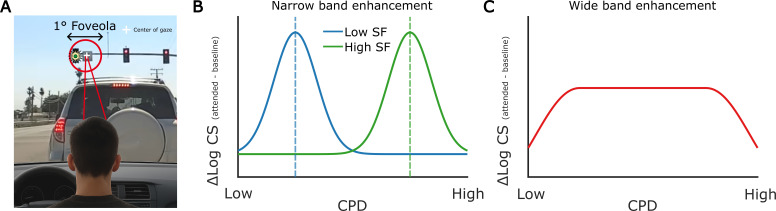
Fine-tuning exogenous attention within the foveola. (**A**) Fine-tuning of exogenous attention within the foveola occurs. For example, when we are looking at a distant traffic light, occupying less than 1° of our visual field, it suddenly turns green, capturing our attention and prompting us to move forward. As a result of the fine-tuning of exogenous attention, contrast sensitivity could be enhanced for a narrow range of spatial frequencies, centered around lower spatial frequencies (blue) or higher spatial frequencies (green) (**B**). On the other hand, contrast sensitivity may be enhanced uniformly across a wide range of spatial frequencies (**C**).

The effects of *extra*foveal attention have been found to differ across SFs: while extrafoveal endogenous attention enhances a broad range of SFs (Lu and Dosher, 2004; Jigo and Carrasco, 2020), extrafoveal exogenous attention selectively enhances high SFs (Carrasco et al., 2006; Barbot et al., 2011; Barbot et al., 2012; Carretié et al., 2012; Jigo and Carrasco, 2020; Fernández et al., 2022), peaking just above the SF characterized by the highest sensitivity at a given eccentricity (Jigo and Carrasco, 2020). Whether fine spatial exogenous attention at the foveal scale modulates visual discrimination similarly is an open question. Generally, fine control of spatial attention at the foveal scale is required when examining fine spatial details, such as reading small text in a book or noticing subtle changes, like a traffic light switching or unexpected pedestrians from afar while driving (Figure 1A). In these tasks, precise allocation of attention likely helps distinguish and recognize individual letters and details. It is possible that in the foveola, exogenous attention modulates a narrow range of SFs, similar to how it operates extrafoveally. However, while humans can resolve SFs up to 30 CPD in the foveola (Intoy and Rucci, 2020; Clark et al., 2022), extrafoveally, SFs above 10 CPD cannot be resolved. Therefore, even if the perceptual enhancement driven by fine spatial attention is limited to a narrow range of lower SFs, the enhanced frequency range may shift toward higher SFs in the foveola compared to what happens extrafoveally (Figure 1B). Alternatively, attention at the foveal scale might preserve its enhancement of low SFs while extending it to high SFs, leading to a broad, rather than narrow, range of modulation (Figure 1C). Any of these scenarios could account for the improvement in orientation discrimination observed in Guzhang et al., 2021.

In the current study, we addressed two main questions. First, does exogenous attention at the foveolar scale enhance visual processing across a narrow or a broader range of SFs? Second, if the enhancement operated within a narrow frequency band, which range of SFs benefits the most from such fine-grained shifts of attention? Addressing these questions is crucial because, while it is now established that covert attention can be selectively shifted even within the central fovea, it remains unclear whether it follows the same *modus operandi* foveally and extrafoveally. If a similar range of SFs is enhanced by exogenous attention in both the foveola and extrafovea, it would suggest that exogenous attention operates similarly across the visual field, regardless of the spatial resolution achievable at different eccentricities. In contrast, if the modulation of SFs differs, it would indicate that the mechanisms of exogenous attention are flexibly tuned in the foveola and adjusted based on the spatial resolution that can be achieved at this scale.

Studying attentional control in the foveola presents unique challenges. Continuous microscopic eye movements during fixation cause constant displacement of the retinal input (Martinez-Conde et al., 2004; Rucci and Poletti, 2015; Krauzlis et al., 2017), making it difficult to limit visual stimulation to the desired eccentricity at this scale. This poses a significant issue when investigating covert attention in the central foveola. To address these challenges, we employed high-precision eye-tracking (Wu et al., 2023) combined with gaze-contingent display control (Santini et al., 2007) to precisely monitor gaze position throughout each trial to ensure that any effects observed are solely due to covert exogenous attention and are not driven by fixational saccades (Hafed and Clark, 2002; Yuval-Greenberg et al., 2014; Shelchkova and Poletti, 2020; Guzhang et al., 2024).

## Results

**Figure 2.**
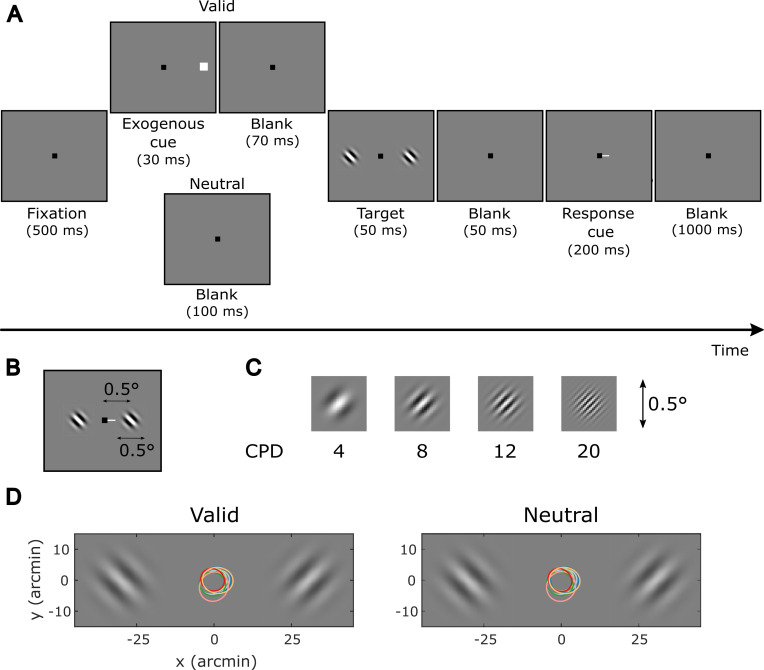
Experimental protocol. (**A**) Trials started with a fixation marker at the center of the monitor. Observers were instructed to maintain fixation at the center throughout the trial. After a brief flash of the exogenous cue to capture observers’ attention, two Gabor patches independently tilted (±45°) were briefly displayed, one on each side of the fixation marker. At the end of the trial, a response cue appeared, and observers had to report the orientation of the stimulus that was previously presented at the cued location. In valid trials, the exogenous cue and response cue indicated the same spatial location. In neutral trials, no exogenous cue was presented. Valid and neutral trials had the same probability of occurrence. (**B**) Size of the stimuli. The Gabor patches had a Gaussian window of 5.4′ standard deviation, creating a 30′ × 30′ visible region. (**C**) Stimuli used in the experiment. Gabor patch of all spatial frequencies tested from 4 to 20 cycles per degree (CPD). (**D**) 68% contour of the gaze probability distribution in valid and neutral conditions during Gabor presentation. Color represents individual observers.

To examine the effects of high-resolution exogenous attention within the fovea on visual discrimination of stimuli at different SFs, we employed a 2AFC visual discrimination task in which observers were asked to discriminate the orientation of a small Gabor patch (30′ × 30′ with an overlaying 5.4′ Gaussian window, tilted ±45°) 30′ from either left or right of the fixation marker when prompted by a response cue (Figure 2A). Note that the Gabor patches used in the current study were much smaller than those typically used in studies probing extrafoveal attention (Rossi and Paradiso, 1995; Gobell and Carrasco, 2005; Herrmann et al., 2010; Jigo and Carrasco, 2020). On each trial, the orientation and phase of the Gabor patch were randomized at each location independently. Eye movements were monitored at high resolution using a digital Dual Purkinje Image eye tracker (Wu et al., 2023) to ensure that observers maintained the center of gaze within a 10′ × 10′ window around the fixation point throughout the trial (Figure 2D). There were no systematic differences in fixation position between valid and neutral conditions, either horizontally (valid: 0.20′ ± 0.66′ SD; neutral: 0.21′ ± 0.63′ SD; p = 0.53) or vertically (valid: –0.52′ ± 0.84′ SD; neutral: –0.48′ ± 0.82′ SD; p = 0.34).

**Figure 2—figure supplement 1.**
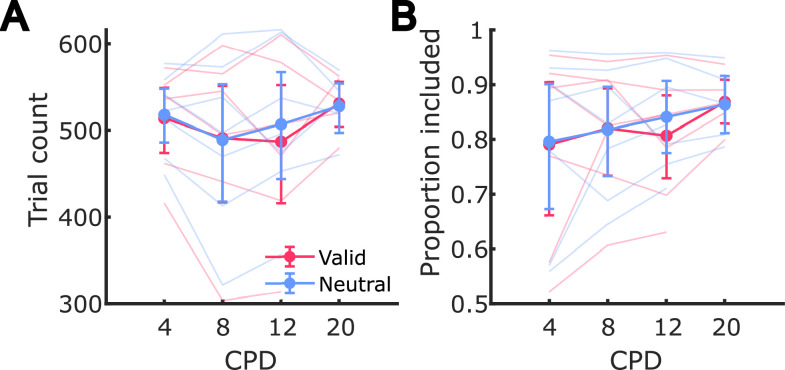
Number of trials included for analysis by condition. (**A**) Number of trials and (**B**) proportion of trials included for analysis in each condition (n = 7). Each line is an observer. Error bars show the bootstrapped 95% confidence intervals.

**Figure 2—figure supplement 2.**
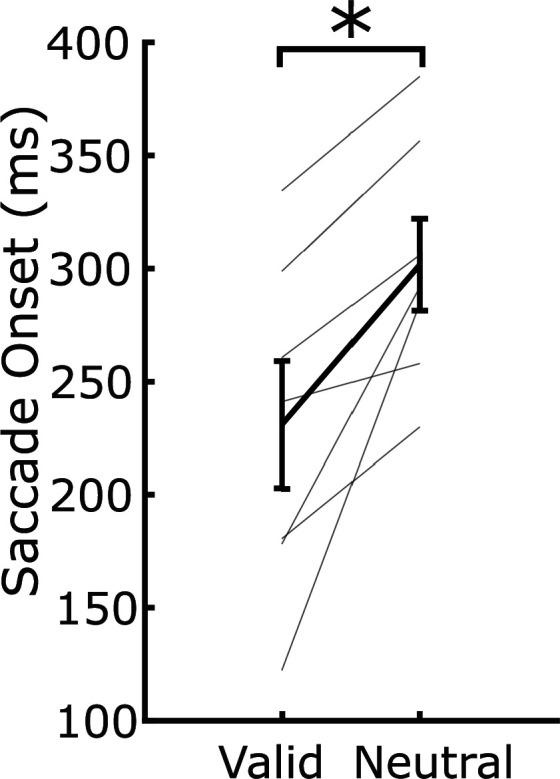
Average saccades onset relative to the response cue onset in valid and neutral conditions. Only saccades that occurred after the fixation period were included in the analysis. The trials presented here were not included in main analyses examining the effects of attention. Lines represent individual observers. Error bars represent ±1 standard error of the mean (SEM). The asterisk indicates a significant difference between conditions (*t*-test, n = 7, p < 0.01).

The experiment included two cueing conditions, valid and neutral, that were randomly interleaved with equal probability within each experimental block. In the valid condition, shifts of exogenous attention were elicited by a small and brief white flash (exogenous cue) presented 100 ms before the target either on the left or the right of the visual field. The cue, with 100% validity, appeared just outside the upcoming target location. In the neutral condition, no exogenous cue was presented.

We tested four SFs, 4, 8, 12, and 20 CPDs, (Figure 2C), ensuring at least one full cycle of modulation within the Gabor patch even at the lowest SF (4 CPD) (Howell and Hess, 1978). The highest SF (20 CPD) is close to the limit of visual resolution at the eccentricity tested here. For each SF tested, an initial threshold contrast was estimated by methods of constant stimuli, then discrimination accuracy was measured at four contrast levels around the initial threshold estimate, and one additional level at the maximum contrast to measure the asymptotic performance (AP; see Methods). The contrast level was kept constant within each experimental block but was randomized across blocks.

**Figure 3.**
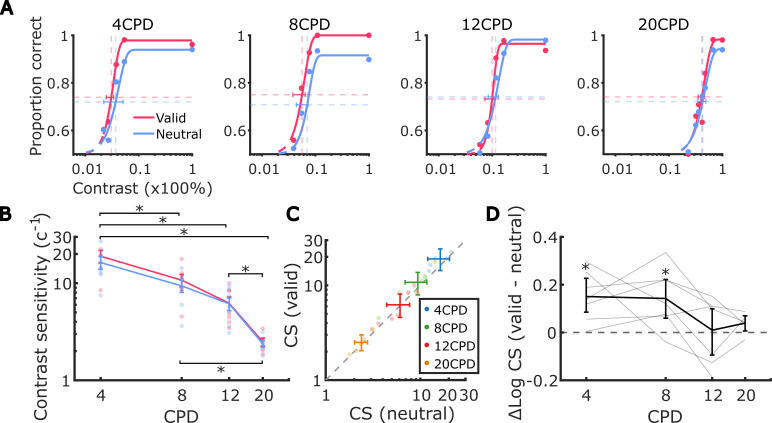
Effect of fine-grained exogenous attention on contrast sensitivity. (**A**) Psychometric functions illustrating example observers’ discrimination accuracy for Gabor patches with spatial frequencies of 4, 8, 12, and 20 cycles per degree (CPD). The size of each dot corresponds to the number of trials included at a specific contrast value. Vertical lines indicate contrast thresholds, while horizontal lines represent the accuracy level midway between chance performance and maximum performance. Error bars around contrast thresholds represent 95% credible intervals. (**B**) Average contrast sensitivity, calculated as the inverse of the contrast threshold, across spatial frequencies in valid and neutral conditions (n = 7). Each dot represents an individual observer. Error bars denote ±1 standard error of the mean (SEM). Asterisks indicate significant post hoc pairwise comparisons between spatial frequencies (*p* < 0.05). (**C**) Average contrast sensitivity in neutral condition against that in valid condition at each spatial frequency (n = 7). Each dot represents an individual observer. Error bars indicate the bootstrapped 95% confidence intervals. (**D**) Average difference in log-scaled contrast sensitivity between valid and neutral conditions across different spatial frequencies (n = 7). Each line corresponds to the log-scaled contrast sensitivities from each observer. Error bars represent the bootstrapped 95% confidence intervals. Asterisks mark post hoc pairwise comparison results between valid and neutral conditions within each spatial frequency (ps < 0.05).

To examine how high-resolution exogenous attention influences performance for stimuli at different SFs, we fitted individual psychometric curves of contrast level vs. discrimination accuracy and estimated contrast thresholds for each cueing condition and SF for each observer (Figure 3A shows the psychometric curves for one example observer with the result of all psychometric curves included in Figure 3—figure supplement 1).

**Figure 3—figure supplement 1.**
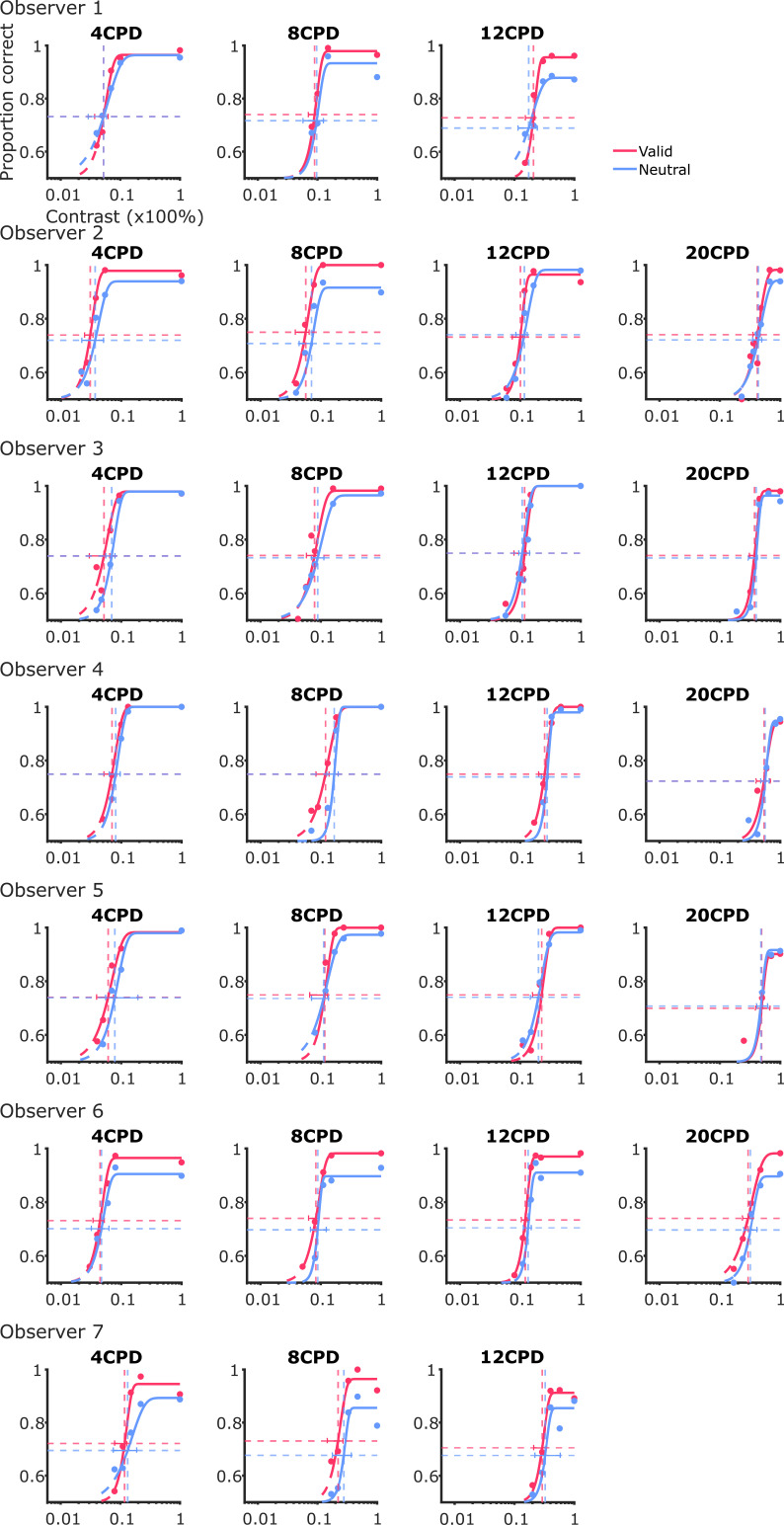
Psychometric Weibull function fits for all observers and conditions (n = 7). Vertical lines indicate contrast thresholds, while horizontal lines represent the accuracy level midway between chance performance and maximum performance. Error bars around contrast thresholds represent 95% credible intervals.

**Figure 3—figure supplement 2.**
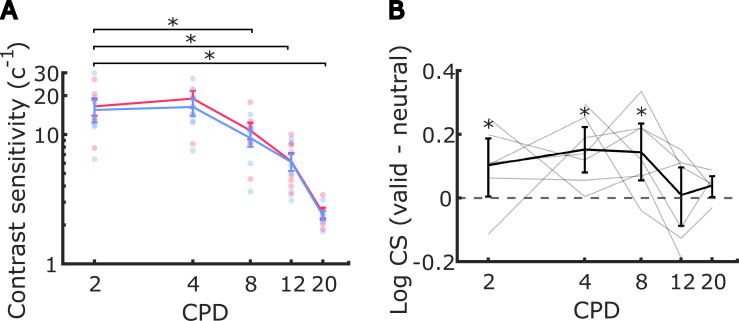
Effect of covert attention on contrast sensitivity at 2 CPD compared to the other spatial frequencies tested. (**A**) Average contrast sensitivity, calculated as the inverse of the contrast threshold, across spatial frequencies, including 2 CPD in valid and neutral conditions (n = 7). Each dot represents an individual observer. Error bars denote ±1 SEM. Asterisks mark significant post hoc pairwise comparisons between 2 CPD and other spatial frequencies (ps < 0.05). (**B**) Average difference in log-scaled contrast sensitivity between valid and neutral conditions across different spatial frequencies (n = 7). Each line corresponds to the log-scaled contrast sensitivities from each observer. Error bars represent the bootstrapped 95% confidence intervals. Asterisks mark post hoc pairwise comparison results between valid and neutral conditions within each spatial frequency (p = 0.04 for valid vs. neutral at 2 CPD; ps > 0.17 for mean gain at 2 CPD vs. other spatial frequencies [SFs]).

**Figure 3—figure supplement 3.**
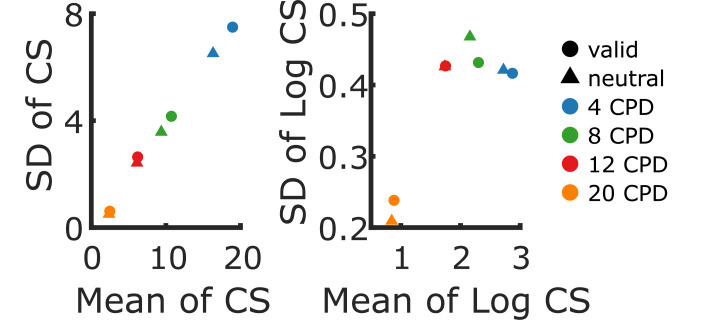
Relation between mean and variability of contrast sensitivity (CS), depending on whether CS is log-transformed (right panel) or not (left panel). Without a log-transform, the mean and standard deviation (SD) of CS are almost perfectly correlated, constituting a strong violation of the homoskedasticity assumption of linear models. For the present data, log-transforming *CS mostly* removes this correlation (except for the 20 CPD condition).

**Figure 3—figure supplement 4.**
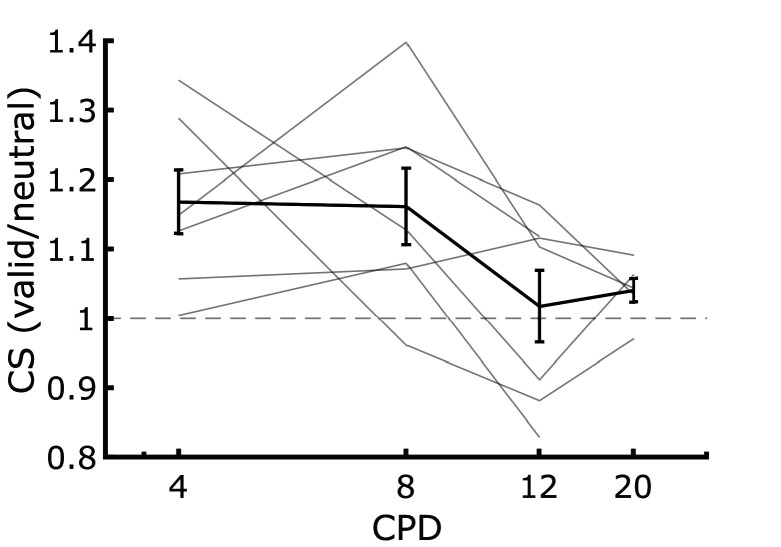
Average attentional benefit calculated as the ratio in contrast sensitivity between valid and neutral conditions (n = 7). Error bars are ±1 SEM.

We first examined the full factorial effects of SF and cueing on CS estimated from the observers’ psychometric functions by fitting a linear mixed-effects regression to the estimates of log-transformed CS at all 8 conditions (4 SF × 2 cueing) across observers. The CS captures the effects on the intercept and/or slope of the psychometric function.

Consistent with the literature (Howell and Hess, 1978; Bex et al., 2009; Lovegrove et al., 1980; Rovamo et al., 1992; Rovamo et al., 1993; Pointer and Hess, 1989), we observed a main effect of SF on CS ($\chi^{2}(3)=65.3$, p < 0.001, Figure 3B; see Appendix 1—table 1 for full model output); CS was highest at 4 CPD and decreased as the SF increased (post hoc pairwise comparisons revealed several significant differences between SFs; post hoc pairwise comparisons of the estimated marginal means, ps ≤ 0.0001 for 4 vs. 8 CPD, 4 vs. 12 CPD, 4 vs. 20 CPD, 8 vs. 20 CPD, 12 vs. 20 CPD). We also observed a significant main effect of attention on CS ($\chi^{2}(1)=7.3$, p < 0.001; $\mathrm{mean}_{\mathrm{valid}}=10.1\pm 7.56$ SD and $\mathrm{mean}_{\mathrm{neutral}}=9.03\pm 6.39$ SD): averaging across all SFs, the valid cueing condition resulted in higher CS (Figure 3B, C). These findings indicate that exogenous attention led to a contrast gain across SFs in the attended subfoveolar region.

Notably, the improvement in CS driven by fine-grained attention was not uniform across SFs (Figure 3D, also visible in Figure 3B, C). We observed a statistically significant interaction between SF and attention ($\chi^{2}(3)=9.3$, p = 0.0258), indicating that contrast gains were selective. Specifically, CS exhibited a contrast gain in the valid condition compared to the neutral condition at lower SFs (4 and 8 CPD) ($\text{mean gain}_{\text{4 CPD}}=2.62\pm 2.13$ SD, Cohen’s $d=0.79$ and $\text{mean gain}_{\text{8 CPD}}=1.36\pm 1.25$ SD, $d=0.75$, ps < 0.004; see Appendix 1—table 2 for all pairwise comparisons). However, the CS gains at higher SFs (12 and 20 CPD) were smaller and were not statistically significant ($\text{mean gain}_{\text{12 CPD}}=0.09\pm 0.91$ SD and $\text{mean gain}_{\text{20 CPD}}=0.11\pm 0.13$ SD, ps > 0.5). In addition to examining the contrast gain within each SF, we also compared the amount of contrast gain within each pair of SFs (see Appendix 1—table 3 for all pairwise comparisons). Post hoc pairwise comparisons revealed that attention modulation did not differ between the two low-mid SFs (4 and 8 CPD, $p > 0.8$) or between the two mid-high SFs (12 and 20 CPD, p > 0.7). Importantly, attention led to a larger enhancement of CS at the low-mid SFs (4 and 8 CPD) compared to 12 CPD ($\Delta\text{mean gain}_{\text{4 CPD - 12 CPD}}=2.53\pm 2.16$ SD, $d=0.65$, and $\Delta\text{mean gain}_{\text{8 CPD - 12 CPD}}=1.27\pm 0.99$ SD, $d=0.62$, ps < 0.025). When comparing the low-mid SFs (4 and 8 CPD) to the highest SF tested (20 CPD), differences in contrast gains were even larger, though these effects were not statistically significant ($\Delta\text{mean gain}_{\text{4 CPD - 20 CPD}}=3.35\pm 1.96$ SD, p = 0.066 and $\Delta\text{mean gain}_{\text{8 CPD - 20 CPD}}=1.45\pm 1.46$ SD, p = 0.084). As detailed in Methods, two observers did not have data for the 20 CPD condition. Statistical power might thus have been reduced for comparisons against this condition.

These results demonstrate that, similar to the selectivity in enhancements in visual periphery, micro-shifts of exogenous attention within the central fovea selectively enhanced CS primarily for low- to mid-range frequencies (4–8 CPD).

In addition to CS, we examined the effects of attention on AP. Research on *extra*foveal vision has returned mixed results with respect to the effects of exogenous attention on AP. It has been found that exogenous attention can enhance both CS (contrast gain) but also AP, a phenomenon known as response gain (Morrone et al., 2002; Morrone et al., 2004; Ling and Carrasco, 2006; Pestilli et al., 2009; Reynolds and Heeger, 2009; Herrmann et al., 2010). However, some studies have found significant effects only on CS, with no notable impact on AP in extrafoveal vision (Cameron et al., 2002; Herrmann et al., 2010; Jigo and Carrasco, 2020). It has been argued that whether covert attention influences contrast gain, response gain, or both, depends on stimulus size, size of the attended area, as well as spatial uncertainty (Herrmann et al., 2010; Wu et al., 2021; Reynolds and Heeger, 2009).

**Figure 4.**
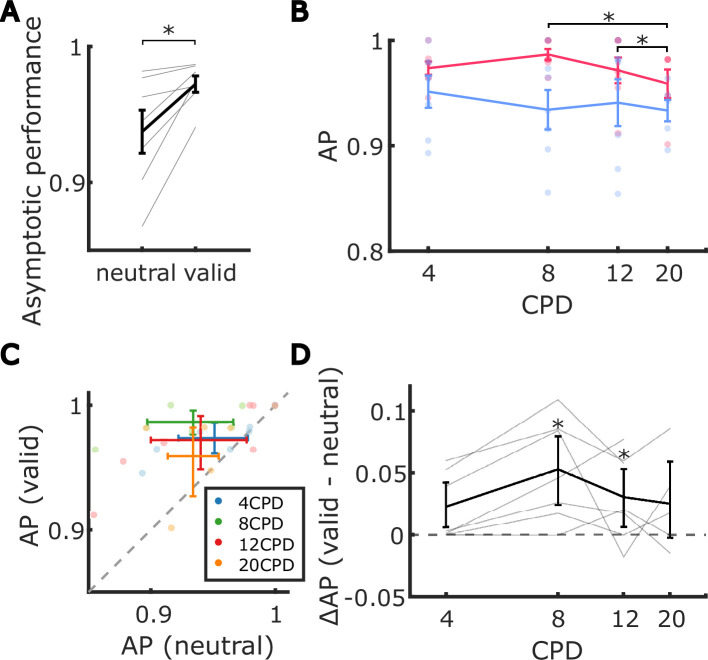
Effect of fine-grained exogenous attention on asymptotic performance. (**A**) Average asymptotic performance, defined as discrimination accuracy at maximum contrast, pooled across spatial frequencies in valid and neutral conditions (n = 7). Each dot represents an individual observer. Error bars denote ±1 SEM. Asterisk indicates a significant main effect of attention (valid vs. neutral) estimated from the GLMM (p < 0.01). (**B**) Average asymptotic performance across spatial frequencies in valid and neutral conditions (n = 7). Each dot represents an individual observer. Error bars denote ±1 SEM. Asterisks mark significant differences in asymptotic performance between pairs of spatial frequencies (ps < 0.05). (**C**) Average asymptotic performance in the neutral condition against that in the valid condition at each spatial frequency (n = 7). Each dot represents an individual observer. Error bars indicate the bootstrapped 95% confidence intervals. (**D**) Average difference in asymptotic performance between valid and neutral conditions across different spatial frequencies (n = 7). Each line corresponds to an individual observer. Error bars indicate the bootstrapped confidence intervals. Asterisks mark post hoc pairwise comparison results between valid and neutral conditions within each spatial frequency (ps < 0.05).

To examine how AP was impacted by the fine-grained shifts of foveal exogenous attention across SFs, we fitted a generalized linear mixed-effects regression to the estimated AP from all 8 conditions of all observers (see Appendix 2—table 1 for full model output). Observers’ ability to discriminate the orientation at full contrast (AP) decreased with increasing frequency (Figure 4B, C), but this change was not significant ($\chi^{2}(3)=7.6$, p = 0.0545) (post hoc pairwise comparisons revealed significant differences in AP only between 8 and 20 CPD (p = 0.015) as well as 12 and 20 CPD (p = 0.048)). The main effect of attention was significant, with overall higher AP in the valid condition compared to the neutral condition ($\chi^{2}(1)=11.9$, p < 0.001; $\mathrm{mean}_{\mathrm{valid}}=0.97\pm 0.03$ SD and $\mathrm{mean}_{\mathrm{neutral}}=0.94\pm 0.04$ SD; see Figure 4A, C). These findings suggest that fine-grained attention resulted in a general response gain, enhancing the ability to discriminate the orientation of high-contrast stimuli.

**Figure 4—figure supplement 1.**
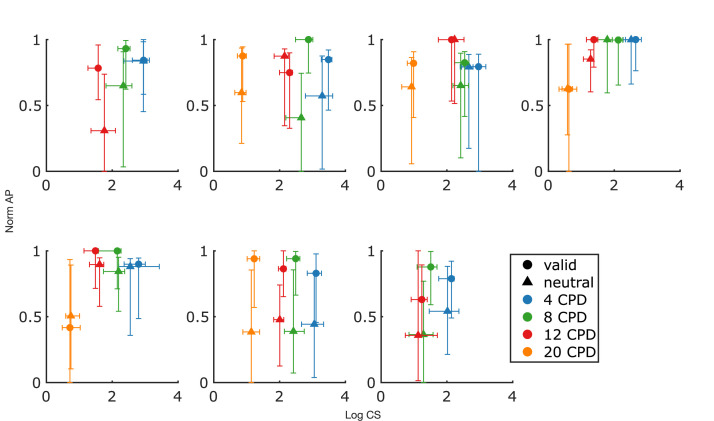
Maximum a posteriori (MAP) estimates (points) and 95% confidence intervals for contrast sensitivity (CS) and asymptotic performance (AP) for each experimental condition across seven observers. Both CS and AP were transformed in the same way as used in our mixed-effects analyses.

Unlike for CS, we did not observe a significant interaction between SF and attention on AP (Figure 4D, $\chi^{2}(3)=5.1$, p > 0.15) (post hoc pairwise comparisons between valid and neutral conditions within each SF revealed that attention significantly increased AP compared to the neutral condition at 8 and 12 CPD (mean gain_8 CPD_ = 0.05 ± 0.04 SD, p < 0.0001 and mean gain_12 CPD_ = 0.03 ± 0.03 SD, p = 0.0108) but not at 4 and 20 CPD (mean gain_4 CPD_ = 0.02 ± 0.03 SD, p = 0.0819 and Δmean gain_20 CPD_ = 0.03 ± 0.04 SD, p = 0.0501; see Appendix 2—table 2 for all pairwise comparisons). When comparing the attentional benefit on AP across pairs of SFs, we found a significant difference (Δmean gain) only between 4 and 8 CPD (p = 0.0240; see Appendix 2—table 3 for all pairwise comparisons)). Therefore, AP exhibited a significant effect of attention alone, with no detected significant effects of SF or of the interaction between SF and attention.

## Discussion

Whereas attention is often believed to be either uniformly allocated at the center of gaze or selectively shifted to locations outside of the central fovea, recent research has shown that humans are also capable of allocating attention within the central fovea in a spatially selective manner, enhancing our ability to perceive fine spatial stimuli (Poletti et al., 2017; Guzhang et al., 2021). Humans can focus processing resources on a specific region of the central fovea, enhancing visual processing within that small area while suppressing processing at other unattended locations just a few arcminutes away.

These findings raise the question of what SFs are enhanced by fine-grained shifts of covert attention within the foveola. In our previous work (Guzhang et al., 2021), observers were asked to perform a coarse orientation (±45°) discrimination task. This experiment did not manipulate SF. Subjectively, however, the stimuli in this task could be distinguished as long as frequency information of more than 3 CPD was available to the observer. The attentional gain we observed in the discrimination task could therefore have resulted from foveal exogenous attention enhancing SFs anywhere above 3 CPD. Thus, it remains unclear which SFs are enhanced when exogenous attention is allocated at the fine scale within the foveola. Additionally, it is unclear whether fine-grained attention in the foveola is governed by the same principles and is modulated similarly to extrafoveal attention, especially considering the stark differences in spatial resolution between the foveola and the rest of the visual field. The high-acuity foveola can resolve SFs up to 30 CPD, whereas just 5° away from the center of gaze, this limit drops to around 10 CPD (Virsu and Rovamo, 1979). Therefore, rather than enhancing the same range of low SFs as for extrafoveal vision, fine-grained foveal attention may shift or extend its enhancement toward higher frequencies in the foveola.

Our findings indicate that fine-grained shifts of covert exogenous attention in the foveola enhance CS within a narrow range of SFs, peaking at low to mid frequencies (4–8 CPD). In particular, we found little or no attentional gain at higher SFs (12–20 CPD), which are closer to the limits of visual resolution at the eccentricity tested (0.3° from the preferred locus of fixation). Whereas enhancements in CS were relatively selective to a narrow band of SFs, overall AP increased as a result of exogenous attention, with no detected dependence on SF. However, it is worth noting that the statistical power to detect the interaction might differ between analyses of CS and analyses of AP, given that the latter tends to involve differences close to its bounds (Bicknell et al., 2025; Jaeger, 2008).

Prior work on coarse exogenous attention shifts between central and peripheral vision has shown that peak attentional benefits for CS occur around 2–4 CPD, with a sizable benefit occurring also at 8 CPD, for a large foveal stimulus (Jigo and Carrasco, 2020). In the present study, the largest attentional gains were observed at the lowest SF tested (4 and 8 CPD). Because of the small stimulus size required to probe fine-grained attention at the foveal scale, we prioritized testing of SFs that yielded at least one full cycle within the stimulus aperture. Nevertheless, we conducted a post hoc evaluation at 2 CPD using the same Gabor size (see Figure 3—figure supplement 2). Because this stimulus contained less than one full cycle, performance may have been compromised (Howell and Hess, 1978). With this caveat in mind, baseline CS was comparable at 2 and 4 CPD and declined at 8 CPD, suggesting a plateau between 2 and 4 CPD. Attention significantly improved CS also at 2 CPD, with contrast gain comparable to that observed at 4 and 8 CPD (see caption of Figure 3—figure supplement 2). Thus, the range of SFs enhanced by fine-grained, exogenously triggered attention closely mirrors that observed when attention is broadly distributed across the fovea. The magnitude of the contrast gain (≈20%) was likewise consistent with prior findings (Jigo and Carrasco, 2020) and slightly larger than that reported by Herrmann et al., 2010 (see Figure 3—figure supplement 4).

### Contrast vs. response gain

Previous studies examining attentional modulation of contrast response functions in extrafoveal vision have reported heterogeneous gain profiles, with some observing predominantly contrast gain (e.g., Cameron et al., 2002; Jigo and Carrasco, 2020) and others reporting a mixture of contrast and response gain (e.g., Ling and Carrasco, 2006; Pestilli et al., 2009). This variability has been attributed to differences in stimulus size and the spatial extent of the attentional field (Reynolds and Heeger, 2009; Herrmann et al., 2010). Within the normalization model of attention, attention multiplicatively scales stimulus drive before normalization and therefore affects both the stimulus drive and, indirectly, the pooled suppressive drive. When the attentional field is small relative to the stimulus, the attended stimulus drive is amplified more than the suppressive drive, producing a multiplicative upward scaling of the contrast response function, with the largest effects at high contrast. On the other hand, when the attentional field is large relative to the stimulus, stimulus and suppressive drive are modulated more proportionally, shifting the effect toward contrast gain. It has also been shown that the effects of endogenous attention manifest as contrast gain, and the effects of exogenous attention can manifest as a mixture between contrast and response gain (Ling and Carrasco, 2006; Pestilli et al., 2009). Hence, the detection of both response and contrast gain in our paradigm, although different from Jigo and Carrasco, 2020, is in line with this expectation.

Further, foveal neurons have smaller integration fields and reduced spatial pooling than peripheral neurons. At the retinal level, classic work on macaque midget ganglion cells showed that receptive fields in the central fovea are extremely small and can be dominated by single-cone input, consistent with minimal spatial pooling at the very center of gaze (Dacey and Petersen, 1992; Dacey, 1993). This characterization seems to be maintained at the LGN (Ramsey et al., 2026). More generally, human EEG studies using steady-state visual evoked potentials, as well as behavioral studies, have shown that surround suppression is stronger in the visual periphery than near the fovea (Xing and Heeger, 2000; Petrov et al., 2005; Vanegas et al., 2015). Although there has been relatively little direct electrophysiological work systematically comparing suppressive zone size or normalization strength between central fovea and peripheral neurons in primate visual cortex, the available evidence raises the possibility that the normalization pool engaged at the foveal scale is smaller and less influential than in extrafoveal vision. In the normalization model of attention, the resulting attention gain profile depends on the balance between the attended stimulus drive and the pooled suppressive drive (Reynolds and Heeger, 2009). If suppressive interactions are indeed weaker within the foveola, then directing attention to a specific foveal locus may amplify the local stimulus drive more than the pooled suppressive drive, thereby favoring response-gain-like effects over contrast-gain-like effects.

Under this interpretation, the detection of response gain in our data may reflect the unusually small integration and suppressive fields engaged by fine-scale attention within the foveola, in contrast with the seemingly more pronounced contrast-gain effects reported in Jigo and Carrasco, 2020. This possibility may also help explain why contrast-gain-like effects were less pronounced at higher SFs in our study, since high SF stimuli are expected to engage smaller receptive fields and even less spatial integration, further reducing the contribution of normalization (Teichert et al., 2007; Serrano-Pedraza et al., 2012). This account—based on post hoc between-study comparisons—remains speculative. Future work will be needed to determine whether fine-scale attention within the fovea is mediated by mechanisms distinct from those operating when attention is distributed more broadly across the central fovea, and whether such differences alter the size or influence of the normalization pool.

### Alternative explanations for the observed effects

While our primary goal was to examine spatially localized, exogenously triggered fine-grained covert attention within the foveola, one could argue that temporal cueing may have contributed to the observed effects. Specifically, even though the timing of the stimulus onset was fixed across trials, the shorter interval between the additional exogenous cue and the target in valid trials could have acted as a temporal warning signal, potentially enhancing performance relative to neutral trials (Duyar et al., 2023). While we cannot entirely rule out temporal cueing effects, their influence is likely limited. Because stimulus timing was fixed and observers completed many trials, the temporal contingency between the cue and stimulus was likely overlearned. Thus, although a minor temporal benefit may have been present in valid trials, the primary attentional advantage observed here is most attributed to spatially localized attentional engagement at the cued location.

Besides the possible contribution of temporal attention, endogenous attention may also have influenced the results. Although our cue was salient and abrupt in onset, and therefore likely triggered an involuntary exogenous shift of attention, its 100% validity could in principle have supported endogenous maintenance following the initial orienting response (Chica et al., 2013). However, endogenous attentional benefits typically emerge on a slower timescale, with onset latencies of approximately 200 ms or longer (Carrasco, 2011). In contrast, the cue–target SOA in our study was only 100 ms, and the target itself was presented for 50 ms. Under these temporal constraints, any endogenous contribution would be expected to arise only after target offset. Thus, the attentional benefits reported here likely reflect rapid, stimulus-driven exogenous mechanisms.

### Future directions

In addition to its perceptual consequences, fine-grained exogenous attention within the foveola may play a preparatory role in oculomotor behavior. When examining the trials that were excluded from main analyses, in which observers happened to perform a saccade following the response cue, we observed a significant reduction in saccade onset latency in valid compared to neutral trials (Figure 2—figure supplement 2). This effect suggests that exogenous attentional deployment within the foveola not only enhances visual sensitivity but may also facilitate the rapid initiation of gaze shifts toward salient events. Although the present task was deliberately constrained, requiring strict fixation during stimulus presentation, this finding hints at a functional coupling between fine-grained exogenous attention within the foveola and the preparation of subsequent eye movements. Future studies using more naturalistic viewing conditions, in which observers are free to move their eyes, will be critical for determining whether this preparatory mechanism serves to efficiently guide microsaccades or saccades toward behaviorally relevant stimuli in everyday vision.

In everyday life, covert exogenous attention is often engaged when a salient stimulus captures our focus. This evolutionarily important mechanism ensures that we continuously monitor our environment for unexpected events and prepare to respond accordingly (Yantis, 1993; Theeuwes, 2010). Our previous work has demonstrated that attentional shifts can also occur locally within the high-acuity foveola. Here, we show that these fine-grained attention shifts function similarly to those in the extrafoveal region, enhancing visual sensitivity to coarse stimulus features. This mechanism is essential for everyday tasks, such as driving or reading. Our findings not only shed light on the functionality of fine-grained covert attention within the foveola but also reinforce the idea that exogenous attention operates under similar principles as extrafoveal vision. Specifically, exogenous attention remains an inflexible mechanism for selective processing—even in the foveola, where higher SF information is available, it does not enhance CS of the finer details but instead prioritizes coarser stimulus features. Functionally, this selective enhancement of CS at low to mid SFs provides a preview of small but salient stimuli located just a few arcminutes from the preferred locus of fixation in everyday tasks. By enhancing CS of these stimuli before direct fixation, this mechanism enables the visual system to rapidly assess their relevance and guide the planning of microsaccades, ensuring efficient and precise shifts of gaze to bring these stimuli into the foveal region for detailed examination.

## Materials and methods

### Observers

Seven human observers in total, 6 emmetropic observers, and 1 observer with 20/20 corrected vision participated in the experiments (4 females, 3 males; age range 18–27 years old). The experiment was approved by the University of Rochester Institutional Review Boards. The experimenter reviewed and explained the material in the consent form to the observers before conducting the experiment. The form was signed only after the observer fully understood the material and voluntarily agreed to take part in the study. Consent was obtained from all observers in the study.

### Stimuli and apparatus

Stimuli were displayed on an LCD monitor (ASUS ROG SWIFT 360 Hz PG259QN) at a refresh rate of 360 Hz and spatial resolution of 1920 × 1080 pixels. Observers performed the task monocularly with their right eye while the left eye was patched. A dental-imprint bite bar and a headrest were used to prevent head movements. Eye movements were recorded with high precision using a custom-made digital Dual Purkinje Image (dDPI) eye tracker, which has a sampling rate of 1 kHz (Wu et al., 2023). The system has an internal noise well below 1′ and a spatial resolution of 1′ (Ko et al., 2016; Wu et al., 2023). Stimuli were rendered using EyeRIS, a custom-developed system that allows flexible gaze-contingent display control (Santini et al., 2007). This system acquires eye movement signals from the eye tracker, processes them in real time, and updates the stimulus on the display according to the desired combination of estimated oculomotor variables.

### Procedure and experimental task

#### Calibration

Every session started with the setup of the bite bar. A magnetized helmet was used to position the observer’s head. When accurate localization of gaze position is necessary, calibration represents an important stage of the experimental procedure, which was performed in two phases: automatic calibration followed by manual calibration. During automatic calibration, observers sequentially fixated on each of the nine points of a 3-by-3 grid, as is customary in all oculomotor experiments. After completing automatic calibration, observers were instructed to perform a manual calibration where they refined the pixel-to-pixel mapping, given by the automatic calibration. To this end, observers fixated again on each of the nine points of the grid while the location of the line of sight was displayed in real time on the screen. Observers used a joypad to correct the predicted gaze location, shifting the real-time display to align with the grid point for each fixation, if necessary. These corrections were then incorporated into the transformation of the gaze position as well. This dual-step calibration procedure allows more accurate localization of gaze position than standard single-step procedures. A similar manual calibration procedure was repeated before each trial but only for the central fixation location to compensate for unpreventable head movements.

#### Experimental task

Observers were instructed to fixate on a central marker (5-by-5 arcminutes) throughout each trial. On valid trials, an exogenous cue, a white square (8-by-8 arcminutes), appeared 500 ms after fixation. The cue appeared for 30 ms at 0.75° eccentricity to the left/right of the fixation marker, with each location occurring randomly with equal probability. The smaller exogenous cue, positioned offset from the Gabor patch, was used to prevent forward masking and ensure clear perception of the Gabor patch. Shortly after the cue disappeared (70 ms), two small Gabor patches (0.5° visible area), tilted ±45°, with a phase of 0° or 90° were shown (50 ms) on the left/right side at 0.5° eccentricity. The tilt of the two Gabor patches was randomly and independently chosen on each trial. The phase was randomly selected on each trial but consistent between the two patches. The SF of the Gabors was 4, 8, 12, or 20 CPD. After the stimulus offset, a response cue was presented, and observers were instructed to report the orientation of the stimulus previously presented at that location. The trial concluded either when observers responded or automatically after 1000 ms if no response was given following the appearance of the response cue. On valid trials, the response cue always indicated the same location as the exogenous cue, making the cue 100% valid. On neutral trials, no exogenous cue was presented, and the response cue indicated one of the two possible locations randomly.

When observers first arrived for the study, they were given task instructions and completed 50 familiarization trials to become accustomed to the setup and the task. Following this, because CS varies considerably across SF and eccentricity (Rovamo et al., 1992), for each SF tested, observers underwent a preliminary session in which an initial estimate of contrast threshold, defined as the contrast needed to achieve 70% discrimination accuracy in the neutral condition. It typically took around 50–100 trials to find the target contrast threshold for each SF. Neither the familiarization trials nor the threshold-estimation trials were included in the final analyses. After the thresholds were obtained, each observer was tested at five different contrast values around the estimated threshold. One of these values included presenting the grating at 100% contrast to obtain a precise estimate for the upper-performance asymptote. The remaining four levels were ±0.075 and ±0.225 log_10_ units from the initial threshold estimate. If the initial estimates were within 0.225 log_10_ units of 100% contrast (i.e., ≥60% contrast), the rest of the four contrast values were −0.6, –0.45, –0.3, and –0.15 log_10_ units compared to the initial estimate (Prins, 2012). Within each experimental session, a single SF was tested, and the corresponding contrast levels were presented in a block design. All five contrast levels for a given SF were tested within a single experimental session. Observers completed 100 trials per contrast level. Each SF was tested twice on two separate days. Therefore, observers completed approximately 4000 trials in total. The order of SF tested was randomized across observers.

Two observers were not tested at 20 CPD because their performance remained at chance level even with gratings at maximal contrast. It is possible given that 20 CPD was near the visual resolution limit at the tested eccentricity.

### Data analysis

#### Eye movements

Only trials with uninterrupted tracking in which the fourth Purkinje image was never eclipsed by the pupil margin were selected for data analysis. Trials in which the gaze was >10′ away from the center position 50 ms before the onset of the exogenous cue *t*_0_ 50 ms after the offset of the target, and trials with blinks, saccades, or microsaccades occurring at any time during the period of interest (50 ms before the onset of the exogenous cue to 200 ms after the offset of the Gabor patches), were discarded. Periods of blinks were automatically detected by the dDPI eye tracker. Eye movements with a minimal amplitude of 30′ and a peak velocity higher than 3°/s were categorized as saccades. Saccades with an amplitude of less than 0.5° (30′) were defined as microsaccades. Saccade amplitude was defined as the vector connecting the point where the speed of the gaze shift grew greater than 3°/s (saccade onset) and the point where it became less than 3°/s (saccade offset). Periods that were not classified as saccades or blinks were labeled as drifts. Observers had 1000 ms to respond, and trials were excluded from further analysis if observers responded too fast (<100 ms) or too slow (>1000 ms), resulting in the exclusion of 0.02% ± 0.02% of the trials. Approximately between 50 and 100 trials per contrast level per cueing condition were retained after filtering. Table 1 summarizes the data remaining for analysis for each condition (see Figure 2—figure supplement 1 for a detailed breakdown by observer).

**Table 1.** Average number and percentage of trials retained for analysis after filtering (mean ± SE across observers) across different spatial frequencies and cueing conditions. Brackets indicate bootstrapped 95% confidence intervals. Observers who were unable to perform the task at 20 CPD were excluded from the 20 CPD trial counts.

	Valid		Neutral	
SF (CPD)	Count	%	Count	%
4	514 [474, 549]	79.1 [66.1, 90.4]	518 [486, 548]	79.6 [67.3, 90.1]
8	491 [417, 551]	82.0 [73.4, 89.3]	489 [418, 553]	81.8 [73.3, 89.6]
12	487 [416, 552]	80.7 [72.9, 88.0]	507 [444, 567]	84.1 [77.5, 90.6]
20	531 [504, 556]	86.8 [82.9, 90.9]	528 [497, 554]	86.4 [81.1, 91.6]

#### Psychometric function fitting

Weibull functions were fitted to the responses of the orientation discrimination task, using the maximum likelihood procedure implemented in the psignifit 4 toolbox (Schütt et al., 2016) for MATLAB. Separate functions were fitted for each combination of observer, attention conditions (attended and neutral), and SF, for a total of 8 psychometric function fits (2 attention conditions × 4 SFs) per observer. Each fit resulted in maximum a posteriori (MAP) estimates for the intercept *α*, slope *β*, threshold *θ*, and lapse rate *λ* of the psychometric function (the guess rate *γ* was set to 0.5, given the 2AFC task). Two estimates were extracted from the MAP estimates to examine the effects of fine-grained exogenous attention across SFs—CS and AP. CS was defined as the inverse of the threshold (the midpoint on the psychometric curve between chance performance and maximum performance). AP was calculated by subtracting the lapse rate from 1, representing the discrimination accuracy at the highest contrast level of the stimuli. In total, this procedure resulted in 52 estimates each of CS and AP (5 observers with 4 SFs × 2 cueing conditions, and 2 observers, who did not complete the 20 CPD condition, with 3 SFs × 2 cueing conditions).

#### Statistical testing

CS and AP were both analyzed with (different types of) mixed-effects regressions. Each of the mixed-effects regressions contained cueing, SF, and their interactions as fixed-effects predictors. Cueing was effect-coded (‘attended’ = 0.5 vs. ‘neutral’ = –0.5), and frequency was coded using sliding difference, comparing the effects for each SF against the next highest SF (4 vs. 8, 8 vs. 12, 12 vs. 20). Following the recommended procedure (Lohse, 2022), we included the maximal possible random effect structure: random intercepts by observer, by unique combination of observer and cueing condition, and by unique combination of observer and SF condition.

CS is a bounded variable with a natural limit in that it cannot be lower than zero. Importantly, the variance of bounded variables tends to systematically decrease as their mean approaches the bound. This violates the assumption of homoskedasticity—the idea that variance should be independent of the mean and thus remain roughly constant across different conditions—an assumption that is shared by widely used statistical methods like *t*-tests, ANOVA (analysis of variance) and linear mixed-effects models (LMMs). When this assumption is violated, it can impact the reliability of statistical conclusions, affecting both Type I errors (false positives) and Type II errors (false negatives) (Jaeger, 2008).

Indeed, we observed a strong positive correlation between the mean and variance of CS: smaller variances for smaller means (Figure 3—figure supplement 3). To address this issue, we log-transformed CS before analyzing it with an LMM using the lmer function from the lme4 package (Bates et al., 2025) in R (R Development Core Team, 2024). This largely mitigated the heteroskedasticity, except potentially in the 20 CPD condition (see Figure 3—figure supplement 3). As a precautionary measure, we verified that all main findings remained unchanged when this condition was excluded. This included the critical interaction between SF and attention ($\chi^{2}(2)=6.6$, p < 0.04), which remained statistically significant.

AP is bounded both at the lower and the upper end (as it cannot be larger than 1, or smaller than the guess rate). Following recommended procedure, we thus normalized AP to the range between 0 and 1, and analyzed it with a mixed-effects Beta model (with a logit link) using the glmmTMB function of the package glmmTMB (McGillycuddy et al., 2025).

Post hoc pairwise comparisons for both mixed-effects analyses were conducted by estimating the relevant marginal means of the fitted mixed-effects regression, using the emmeans package (Lenth et al., 2025) in R. Cohen’s *d* (Cohen, 1988) of the pairwise differences was computed using the function t_to_d from the package effectsize (Ben-Shachar et al., 2020) in R.

#### Power analysis

Our initial sample size estimate was an approximation based on our previous study using a similar design to examine exogenous attention during high-acuity stimulus discrimination within the foveola (Guzhang et al., 2021). To formalize this estimate, we conducted a post hoc power analysis following approaches used in prior work on *extra*foveal attention (Jigo and Carrasco, 2020) to estimate the sample size required to detect attentional effects on contrast gain. We assumed effect sizes comparable to those observed in our previous study (Guzhang et al., 2021). Specifically, we followed a bootstrap approach (McConnell and Vera-Hernández, 2015; Jigo and Carrasco, 2020) in which data from two to twelve observers were randomly sampled with replacement from Guzhang et al., 2021. For each resampled dataset, we conducted a one-way repeated-measures ANOVA with attention as a within-subject factor. This procedure was repeated 10,000 times, and a distribution of p-values was constructed for the main effect of attention. Statistical power was estimated as the proportion of iterations yielding a significant main effect (p < 0.05) for each sample size. The results indicated that a sample size of five observers was sufficient to achieve statistical power greater than 80% for detecting the main effect of attention. Based on this simulation, the sample size of seven observers used in the present study should provide adequate statistical power.

We conducted additional post hoc power analyses to evaluate the power of our design to detect main effects and their interactions, using the *simr* function of the package *simr* in R. We estimated statistical power for mixed-effects models through model-based simulation by generating synthetic datasets based on the fixed and random effects structure of the fitted model, preserving the observed effect sizes and variance components. For each simulated dataset, the model was refitted, and the effect of interest was tested. By repeating this procedure 501 times across different sample sizes, power was estimated as the proportion of simulations in which the effect was statistically significant (p < 0.05). Consistent with the bootstrap power analysis reported above, the results show that our study had high power (>95%) to detect the main effects of attention and SF, and moderate power (>65%) to detect the interaction. Because classical post hoc power analyses tend to be circular and anti-conservative (Quach et al., 2022; Heinsberg and Weeks, 2022), we also used the same approach while allowing for potential overestimation of effect sizes and underestimation of variance components. Under deliberately conservative assumptions, specifically, effect sizes reduced to 75% of those observed and variance components increased by 25%, power to detect the main effects remained above 85%, while power to detect the interaction (which was nevertheless observed) was approximately 50%.

Below, we summarize the key considerations for interpreting our results.

#### Limitations

Here, we analyzed log-transformed CS and AP in two separate analyses, each conducted over the observer-level estimates for each condition. We did so both (1) because this approach remains the standard in psychophysics—including in research on the role of covert attention (Cameron et al., 2002; Pestilli and Carrasco, 2005; Herrmann et al., 2010; Li et al., 2021)—and (2) because the alternative would have required fitting mixed-effects *trial-level* psychometric models to the combined data from all conditions and observers (an approach that is computationally demanding, and has not yet been broadly validated). The approach taken here and in prior work on extrafoveal attention does, however, have several known limitations, some of which might be of particular relevance to questions about the effects of attention. We summarize these potential downsides here, so that they can be considered in the interpretation of our results, and addressed in future work.

First, reducing each observer’s performance in a given condition to the best-fitting psychometric parameters (e.g., sensitivity or AP) ignores the uncertainty associated with those estimates. Subsequent repeated-measures ANOVAs or, in our case, mixed-effects regression analyses performed on these point estimates cannot take advantage of the information present in the trial-level data. As a result, two parameter estimates with very different levels of uncertainty (see Figure 4—figure supplement 1) may have similar influence on the analysis despite substantial differences in precision. Importantly, the precision of psychometric estimates can vary across observers and conditions because the amount and distribution of usable data differ, and because the sampled stimuli may probe different portions of the psychometric function. Ultimately, failure to adequately account for uncertainty can lead to miscalibrated inference, increasing the risk of both Type I and II errors.

Second, the standard approach ignores the interdependence among psychometric parameters. In practice, the same data can often be fit nearly equally well by different combinations of parameter values, for example, a steeper slope paired with a lower lapse rate, or a shallower slope paired with a higher asymptote. In particular, when performance fails to adequately constrain the upper asymptote, parameters such as threshold, slope, and lapse or AP can become difficult to disentangle. When only the best-fitting parameter values are carried forward, all information about such covariance between parameters is lost. As a result, apparent differences in whether attention affects contrast gain or response gain may partly reflect parameter tradeoffs rather than genuine differences across studies.

Future research could employ alternative analysis approaches. In particular, it is now possible to fit mixed-effects psychometric models to the trial-level data from all conditions and all observers (Prins, 2024; Tan and Jaeger, 2025). While this approach is computationally more demanding and requires familiarity with nonlinear mixed-effects modeling, it allows statistical tests that avoid the downsides described above.

## Data Availability

The dataset and analysis code associated with this work are available on Open Science Framework (OSF) https://doi.org/10.17605/OSF.IO/PG7HD. The following dataset was generated: GuzhangY JaegerTF PolettiM 2026Frequency-dependent modulation of foveal contrast sensitivity by fine-scale exogenously triggered attentionOpen Science Framework10.17605/OSF.IO/PG7HDPMC1331369342371699

## Additional files

MDAR checklist

## Appendix 1

### Effects of attention and spatial frequency on contrast sensitivity

**Appendix 1—table 1.** Linear mixed-effects model predicting log-transformed contrast sensitivity from attention, spatial frequency, and their interaction.

Fixed effect	Estimate	SE	95% CI	df	*t*	p
Intercept	1.879	0.148	[1.583, 2.175]	5.96	12.73	<0.001
Attention (valid vs. neutral)	0.084	0.028	[0.029, 0.139]	5.54	2.99	0.027
Frequency (8 vs. 4)	–0.566	0.083	[–0.729, –0.403]	15.95	–6.85	<0.001
Frequency (12 vs. 8)	–0.485	0.083	[–0.648, –0.322]	15.95	–5.87	<0.001
Frequency (20 vs. 12)	–0.997	0.093	[–1.179, –0.815]	16.04	–10.73	<0.001
Attention × frequency (8 vs. 4)	–0.008	0.054	[–0.114, 0.098]	15.53	–0.14	0.889
Attention × frequency (12 vs. 8)	–0.134	0.054	[–0.240, –0.028]	15.53	–2.48	0.025
Attention × frequency (20 vs. 12)	0.022	0.060	[–0.096, 0.140]	16.46	0.37	0.714

#### Random effects

Observer (Intercept): SD = 0.3809

Observer:Attention (Intercept): SD = 0.0362

Observer:Frequency (Intercept): SD = 0.1461

Residual: SD = 0.0715

REML criterion = –20.4; 52 observations; Observer $n=7$.

**Appendix 1—table 2.** Simple effects of attention on contrast sensitivity at each spatial frequency (SF) and their effect sizes in Cohen’s *d*.

SF	Estimate	SE	95% CI	df	*t*	p	Cohen’s *d*	Estimate (ratio)
4	0.151	0.043	[0.061, 0.240]	19.76	3.52	0.002	0.79	1.16
8	0.143	0.043	[0.054, 0.233]	19.76	3.34	0.003	0.75	1.15
12	0.009	0.043	[–0.080, 0.099]	19.76	0.22	0.830	0.05	1.01
20	0.032	0.051	[–0.074, 0.137]	21.37	0.62	0.540	0.13	1.03

**Appendix 1—table 3.** Interaction contrasts comparing attention effects between spatial frequency (SF) pairs and their effect sizes in Cohen’s *d*.

SF pair	Estimate	SE	95% CI	df	*t*	p	Cohen’s *d*
4–8	0.008	0.054	[–0.107, 0.122]	16.0	0.14	0.889	0.04
4–12	0.141	0.054	[0.027, 0.256]	16.0	2.62	0.019	0.65
4–20	0.119	0.061	[–0.009, 0.247]	16.9	1.97	0.066	0.48
8–12	0.134	0.054	[0.019, 0.248]	16.0	2.48	0.025	0.62
8–20	0.111	0.061	[–0.016, 0.239]	16.9	1.84	0.084	0.45
12–20	–0.022	0.061	[–0.150, 0.106]	16.9	–0.37	0.717	–0.09

## Appendix 2

### Effects of attention and spatial frequency on asymptotic performance

**Appendix 2—table 1.** Generalized linear mixed-effects model predicting normalized asymptotic performance from attention, spatial frequency, and their interaction.

Fixed effect	Estimate	SE	95% CI	*z*	p
Intercept	2.676	0.349	[1.99, 3.36]	7.67	<0.001
Attention (valid vs. neutral)	0.960	0.178	[0.61, 1.31]	5.41	<0.001
Frequency (8 vs. 4)	0.289	0.353	[–0.40, 0.98]	0.82	0.414
Frequency (12 vs. 8)	–0.181	0.351	[–0.87, 0.51]	–0.52	0.606
Frequency (20 vs. 12)	–1.020	0.394	[–1.79, –0.25]	–2.59	0.010
Attention × frequency (8 vs. 4)	1.127	0.499	[0.15, 2.11]	2.26	0.024
Attention × frequency (12 vs. 8)	–0.820	0.506	[–1.81, 0.17]	–1.62	0.105
Attention × frequency (20 vs. 12)	–0.167	0.493	[–1.13, 0.80]	–0.34	0.734

#### Random effects

Observer (Intercept): SD = 0.8427

Observer:Attention (Intercept): SD = 3.584e-05

Observer:Frequency (Intercept): SD = 0.4493

Model family: beta (logit link); Dispersion parameter = 34.6; AIC = –184.9;

52 observations; Observer $n=7$.

**Appendix 2—table 2.** Simple effects of attention on asymptotic performance at each spatial frequency (SF) and their effect sizes in Cohen’s *d*.

SF	Estimate	SE	95% CI	*z*	p	Cohen’s *d*	Estimate (prob. diff.)
4	0.566	0.325	[–0.072, 1.204]	1.74	0.082	0.39	0.030
8	1.693	0.392	[0.925, 2.461]	4.32	<0.001	0.97	0.077
12	0.874	0.329	[0.230, 1.518]	2.66	0.008	0.60	0.044
20	0.707	0.361	[0.000, 1.414]	1.96	0.050	0.42	0.081

**Appendix 2—table 3.** Interaction contrasts comparing attention effects between spatial frequency (SF) pairs on asymptotic performance and their effect sizes in Cohen’s *d*.

SF pair	Estimate	SE	95% CI	*z*	p	Cohen’s *d*
4–8	–1.127	0.499	[–2.106, –0.149]	–2.26	0.024	–0.56
4–12	–0.308	0.460	[–1.209, 0.593]	–0.67	0.503	–0.17
4–20	–0.140	0.488	[–1.098, 0.817]	–0.29	0.774	–0.07
8–12	0.820	0.506	[–0.173, 1.812]	1.62	0.105	0.40
8–20	0.987	0.537	[–0.066, 2.040]	1.84	0.066	0.45
12–20	0.167	0.493	[–0.798, 1.133]	0.34	0.734	0.08
