# Supplementary material for: Frequency-dependent modulation of foveal contrast sensitivity by fine-scale exogenously triggered attention
Source: eLife. 2026 Jun 29;14:RP108788. doi: 10.7554/eLife.108788 (PMC13313693; doi:10.7554/eLife.108788)
Supplement: Peer review history

# Editor report: eLife Assessment

**DOI:** 10.7554/eLife.108788.3.sa0

**Authors:** Emilio Salinas

**Affiliations:** Wake Forest University School of Medicine United States

**Keywords:** Compelling, Important

This study explores how exogenous attention operates at the finest spatial scale of vision, within the foveola - a topic that has not been previously explored but is of interest to visual neuroscientists. The question is **important** for understanding how attention shapes perception, and how it differs between the periphery and the central regions of highest visual acuity. The evidence indicating that attention near the fovea preferentially enhances low spatial frequencies is **compelling**, as shown by carefully designed experiments with state-of-the-art eye tracking to monitor attended locations just a few tens of minutes of arc away from the fixation target.

# Referee report: Reviewer #1 (Public review)

**DOI:** 10.7554/eLife.108788.3.sa1

[Editors' note: this version has been assessed by the Reviewing Editor without further input from the original reviewers. The authors have addressed the weaknesses noted above, which were raised in the previous round of review.]

Summary:

The manuscript investigates how exogenous attention modulates spatial frequency sensitivity within the foveola. Using high-precision eye-tracking and gaze-contingent stimulus control, the authors show that exogenous attention selectively improves contrast sensitivity for low- to mid-range spatial frequencies (4-8 cycles/degree), but not for higher frequencies (12-20 CPD). In contrast, improvements in asymptotic performance at the highest contrast levels occur across all spatial frequencies. These results suggest that, even within the foveola, exogenous attention operates through a mechanism similar to that observed in peripheral vision, preferentially enhancing lower spatial frequencies.

Strengths:

The study shows strong methodological rigor. Eye position was carefully controlled, and the stimulus generation and calibration were highly precise. The authors also situate their work well within the existing literature, providing a clear rationale for examining the fine-grained effects of exogenous attention within the foveola. The combination of high spatial precision, gaze-contingent presentation, and detailed modeling makes this a valuable technical contribution.

Weaknesses:

The manipulation of attention raises some interpretive concerns. Clarifying this issue, together with additional detail about statistics, participant profiles, other methodological elements, and further discussion in relation to oculomotor control in general, could broaden the impact of the findings.

# Referee report: Reviewer #2 (Public review)

**DOI:** 10.7554/eLife.108788.3.sa2

Summary:

This study aims to test whether foveal and non-foveal vision share the same mechanisms for endogenous attention. Specifically, they aim to test whether they can replicate at the foveola previous results regarding the effects of exogenous attention for different spatial frequencies.

Strengths:

Monitoring the exact place where the gaze is located at this scale requires very precise eye-tracking methods and accurate and stable calibration. This study uses state-of-the-art methods to achieve this goal. The study builds on many other studies that show similarities between foveal vision and non-foveal vision, adding more data supporting this parallel.

Weaknesses:

The study lacks a discussion of the strength of the effect and how it relates to previous studies done away from the fovea. It would be valuable to know if not just the range of frequencies, but the size of the effect is also comparable.

# Referee report: Reviewer #3 (Public review)

**DOI:** 10.7554/eLife.108788.3.sa3

Summary:

This paper explores how spatial attention affects foveal information processing across different spatial frequencies. The results indicate that exogenously directed attention enhances contrast sensitivity for low- to mid-range spatial frequencies (4-8 CPD), with no significant benefits for higher spatial frequencies (12-20 CPD). However, asymptotic performance increased as a result of spatial attention independently of spatial frequency.

Strengths:

The strengths of this article lie in its methodological approach, which combines a psychophysical experiment with precise control over the information presented in the foveola.

Weaknesses:

The authors acknowledge that they used the standard approach of analyzing observer-averaged data, but recognize that this method has limitations: it ignores the uncertainty associated with parameter estimates and the relationships between different parameters of the psychometric model. This may affect the interpretation of attentional effects. In the future, mixed-effects models at the trial level could overcome these limitations.

# Author comment: Author response

**DOI:** 10.7554/eLife.108788.3.sa4

**Authors:** Yue Guzhang, T Florian Jaeger, Martina Poletti

**Affiliations:** University of Rochester Rochester United States; University of Rochester Rochester United States; University of Rochester Medical Center New York United States

The following is the authors’ response to the original reviews.

**eLife Assessment**

This study explores how exogenous attention operates at the finest spatial scale of vision, within the foveola - a topic that has not been previously explored. The question is important for understanding how attention shapes perception, and how it differs between the periphery and the central regions of highest visual acuity. The evidence is compelling, as shown by carefully designed experiments with state-of-the-art eye tracking to monitor attended locations just a few tens of minutes of arc away from the fixation target, but additional clarification regarding analyses and implications for vision and oculomotor control would broaden the impact of the study.

We thank the editors and reviewers for their thorough evaluation of our work. We have carefully revised the manuscript and substantially reworked the Discussion to address all of the points raised, eliminate redundancies, streamline the text, and clarify the implications of our findings for vision and oculomotor control. We have also expanded the documentation of our power analyses and conducted the additional analyses requested by the reviewers. Our point-by-point responses are provided.

**Public Reviews:**

**Reviewer #1 (Public review):**

Summary:

The manuscript investigates how exogenous attention modulates spatial frequency sensitivity within the foveola. Using high-precision eye-tracking and gaze-contingent stimulus control, the authors show that exogenous attention selectively improves contrast sensitivity for low- to midrange spatial frequencies (4-8 cycles/degree), but not for higher frequencies (12-20 CPD). In contrast, improvements in asymptotic performance at the highest contrast levels occur across all spatial frequencies. These results suggest that, even within the foveola, exogenous attention operates through a mechanism similar to that observed in peripheral vision, preferentially enhancing lower spatial frequencies.

Strengths:

The study shows strong methodological rigor. Eye position was carefully controlled, and the stimulus generation and calibration were highly precise. The authors also situate their work well within the existing literature, providing a clear rationale for examining the fine-grained effects of exogenous attention within the foveola. The combination of high spatial precision, gazecontingent presentation, and detailed modeling makes this a valuable technical contribution.

Weaknesses:

The manipulation of attention raises some interpretive concerns. Clarifying this issue, together with additional detail about statistics, participant profiles, other methodological elements, and further discussion in relation to oculomotor control in general, could broaden the impact of the findings.

We thank the reviewer for the helpful comments. In the Discussion, we have now considered additional factors that could have contributed to the observed attentional effects. First, the exogenous cue might have functioned as a temporal warning signal. However, the interval between cue and stimulus onset was fixed across trials, meaning that the cue did not provide temporal information beyond what participants could already anticipate. Furthermore, participants completed a large number of trials (≥ 4000), making it highly likely that the temporal relationship between trial onset and target onset was overlearned. These considerations indicate that the observed benefit in the valid condition was predominantly attributable to spatial reorienting induced by the cue, rather than to differences in the temporal predictability of the target across conditions.

Another possibility is that the 100% validity of the exogenous cue could potentially have promoted endogenous attentional engagement. Yet, several characteristics of our task strongly limited the extent to which such endogenous engagement could meaningfully influence performance. Endogenous attentional benefits typically emerge only after ~150-200 ms (Posner & Petersen, 1990; Carrasco, 2011), whereas our cue-target SOA was 100 ms, and the target remained visible for only 50 ms. Under these temporal constraints, any voluntary, slow endogenous enhancement would primarily occur after the stimulus offset. Thus, although endogenous maintenance is theoretically possible given the cue’s validity, it is unlikely to have substantially contributed to the observed attentional benefits in our task.

Regarding the points on statistical reporting and participant details, we followed the reviewer’s suggestions by adding post hoc power analyses and providing more comprehensive reporting of the linear model outputs (see Appendices 1 and 2). We also expanded the description of the training procedures conducted with participants prior to formal data collection in the Methods section.

We appreciate the reviewer for raising the important question of how our findings may relate to oculomotor control. To address this, we analyzed trials excluded from the manuscript due to saccades. This analysis revealed that saccade latencies were shorter in the valid condition than in the neutral condition (see Figure 2 — Supplementary Figure 2). This earlier saccade onset may reflect exogenously triggered preparatory activity in the oculomotor system in response to the salient cue. Future studies are needed to examine whether this preparatory mechanism serves to efficiently guide microsaccades or saccades toward behaviorally relevant stimuli in everyday vision. We have incorporated this point into the Discussion, highlighting a potential mechanistic link between exogenous attention and oculomotor behavior.

**Reviewer #2 (Public review):**

Summary:

This study aims to test whether foveal and non-foveal vision share the same mechanisms for endogenous attention. Specifically, they aim to test whether they can replicate at the foveola previous results regarding the effects of exogenous attention for different spatial frequencies.

Strengths:

Monitoring the exact place where the gaze is located at this scale requires very precise eyetracking methods and accurate and stable calibration. This study uses state-of-the-art methods to achieve this goal. The study builds on many other studies that show similarities between foveal vision and non-foveal vision, adding more data supporting this parallel.

Weaknesses:

The study lacks a discussion of the strength of the effect and how it relates to previous studies done away from the fovea. It would be valuable to know if not just the range of frequencies, but the size of the effect is also comparable.

We thank the reviewer for raising these important issues. In response, we have expanded the Discussion to link our findings to prior work. First, we included a direct comparison of our effect sizes with those reported in previous studies. This analysis revealed that our effect sizes are highly comparable to those earlier studies (see Figure 3 — Supplementary Figure 4). Second, we contextualized our findings within the popular framework of normalization model of attention in the Discussion. We detected a mixture of contrast and response gain effects, consistent with predictions from the normalization framework given our experimental design. Finally, we extended the Discussion to consider potential underlying neural mechanisms. Specifically, we suggested that differences in attentional modulation, particularly the manifestation in response gain vs. contrast gain between the fovea and extrafovea, may reflect distinct characteristics of foveal neurons relative to those in extrafoveal regions.

**Reviewer #3 (Public review):**

Summary:

This paper explores how spatial attention affects foveal information processing across different spatial frequencies. The results indicate that exogenously directed attention enhances contrast sensitivity for low- to mid-range spatial frequencies (4-8 CPD), with no significant benefits for higher spatial frequencies (12-20 CPD). However, asymptotic performance increased as a result of spatial attention independently of spatial frequency.

Strengths:

The strengths of this article lie in its methodological approach, which combines a psychophysical experiment with precise control over the information presented in the foveola.

Weaknesses:

The authors acknowledge that they used the standard approach of analyzing observeraveraged data, but recognize that this method has limitations: it ignores the uncertainty associated with parameter estimates and the relationships between different parameters of the psychometric model. This may affect the interpretation of attentional effects. In the future, mixed-effects models at the trial level could overcome these limitations.

We thank the reviewer for this comment. Our Methods section continues to transparently discuss these limitations, as well as the fact that these limitations are shared with most published studies in psychophysics. Additionally, we now include measures of uncertainty for all key effects (see Appendices 1 and 2), and we have reported effect sizes throughout the Results section. Finally, we have added post hoc power analyses to the Methods. Following previous approaches to power calculation for related experiments, we found that our study was sufficiently powered to detect the main effect of attention and had moderate power to detect the interaction between attention and spatial frequency.

**Recommendations for the authors:**

**Reviewer #1 (Recommendations for the authors):**

(1) The manipulation of attention raises some interpretive concerns. Since only valid and neutral cue conditions were included, the results might reflect differences in temporal predictability rather than true spatial reorienting of attention. In other words, the valid cue could act mainly as a temporal warning signal that reduces uncertainty about stimulus onset. Without invalid trials or a non-predictive control cue, it remains difficult to separate spatial and temporal contributions to exogenous attention.

We thank the reviewer for raising this point. In this regard, we would like to clarify that there was no temporal uncertainty in stimulus onset: across all conditions and trial types, the stimulus was presented at the same time relative to the start of the trial, i.e., 600 ms after the start. Yet, we acknowledge that the shorter temporal proximity between the cue and stimulus in valid trials could serve as an additional temporal warning signal, potentially conferring an advantage relative to the neutral condition. While we cannot completely rule out a contribution of such temporal cueing within the constraints of the current experimental design, we believe its impact was limited. Specifically, the fixed cue-stimulus interval reduced the cue’s ability to convey additional temporal information. Furthermore, observers completed a large number of trials (≥4000), and the temporal contingency between trial onset and target onset was likely overlearned. Taken together, these considerations indicate that the observed benefit in the valid condition was predominantly attributable to spatial reorienting induced by the cue, rather than to differences in the temporal predictability of the target across conditions. We now mention this in the revised Discussion (lines 309-318).

We recognized that the original Figure 2 illustrating the experimental paradigm may have caused confusion regarding the timing structure of the task. We have therefore updated the figure to more explicitly illustrate the trial timeline in both conditions.

(2) The reported effects seem small, and no power analysis is provided. With only seven participants, the study may not have enough statistical power to confirm that the observed differences are reliable or generalizable. Although the technical precision in gaze and stimulus control is impressive, it cannot offset the limitations of a small sample. The authors should include effect size estimates, confidence intervals, and ideally a post-hoc power analysis.

The statistical results are reported only as χ² values from model comparisons, which do not show the direction or size of the effects. For clarity and transparency, these tests should be accompanied by fixed-effect estimates with their standard errors and confidence intervals, so readers can better assess both the reliability and perceptual relevance of the findings.

The reviewer raised several important points regarding the study's statistical rigor.

In the revised manuscript, we now report effect size estimates (Cohen’s d) in the Results section and Appendices. Effect sizes were in the medium-to-large range, including the effect of attention on contrast sensitivity at 4 and 8 CPD, and the difference in attentional benefit on contrast sensitivity between 4 and 12 CPD and between 8 and 12 CPD. We have also included the full model outputs, including standard errors and confidence intervals, in the Appendices.

The sample size for the current study was determined based on the magnitude of the attentional effects observed in our previous work (Guzhang et al., 2021). The experimental design and dependent measures were highly similar across the two studies, and the prior study revealed a robust effect, which accounted for a substantial proportion of within-observer variance in a tightly controlled repeated-measures design.

We have revised the manuscript, adding bootstrap-based power estimates, following the procedure described by Jigo and Carrasco (2020), using data from Guzhang et al. (2021). Assuming the effect size in our current study would be comparable to the prior one, 2 to 12 observers were randomly sampled with replacement, and a one-way repeated-measures ANOVA with attention as the main factor was used. This procedure was repeated 10,000 times, and power was estimated as the proportion of iterations yielding a significant main effect for each sample size. The results of this analysis indicate that a sample size of five observers would have been sufficient to achieve approximately 80% power to detect the main effect of attention in the prior study. Based on these estimates, the sample size used in the current study (seven observers) is adequately powered.

We also conducted a post hoc power analysis to evaluate the power of our design to detect the main effects and their interaction. It was performed using the R package *simr*, which estimates statistical power for mixed-effects models through model-based simulation. Specifically, *simr* generated datasets based on the fixed- and random-effect structure of the fitted model, preserving the observed effect sizes and variance components. For each simulated dataset, the model was refit, and the effect of interest was tested. By repeating this procedure 501 times across different sample sizes, power was estimated as the proportion of simulations in which the effect was statistically significant. Based on these post hoc simulations, we estimated that our study had high power (>95%) to detect the main effects and moderate power (>65%) to detect the interaction. Although the estimated power for the interaction was lower than for the main effects, the observed effect size was substantial (as indexed by Cohen’s *d*), indicating that the interaction was not trivially small.

We now describe these analyses in lines 501-532 in the Methods section.

(3) The task seems quite demanding, requiring fine spatial discrimination, very small stimuli, and head stabilization with a bite bar. It is not clear whether participants were naïve or experienced observers. If they had prior psychophysical training, practice effects could have influenced the results, particularly given the lack of invalid trials. The manuscript would benefit from clarifying participants' experience level and describing any training or familiarization procedures.

We appreciate the reviewer’s concern regarding potential training effects. All observers had prior experience with similar tasks, but were naïve to the scope of this study. Each participant underwent an initial familiarization phase of approximately 50 trials with the experimental setup of this study. They then completed an additional ~50 trials to estimate their individual contrast thresholds per spatial frequency level before we proceeded with data collection at the five predefined contrast levels.

Based on our experience, we have found that, for experiments similar to the one described here, observers quickly adapt to the setup and are generally able to maintain reliable fixation and stable performance, even during the initial training phase. In addition, each participant completed approximately 400 trials before the data collection started. Even observers who began the session with no prior experience would have become practiced with the setup by the time the actual data-collection phase started, during which ~4000 trials were collected per observer. Therefore, whether an observer participated in previous experiments is unlikely to meaningfully affect the results, as the large number of trials ensures comparable levels of task familiarity across individuals.

Crucially, valid and neutral trials were interleaved throughout the session. Any general learning or practice would therefore influence both conditions equally. Despite this, we still observed clear performance improvements in the valid condition relative to the neutral condition, indicating that the observed benefits cannot be attributed solely to practice and reflect an attentional enhancement. We have added elaboration on the training procedures in Methods (lines 411-429).

Finally, we recognize that the lack of invalid trials may raise concerns given our 100% spatially predictive cue, as noted in Reviewer 3’s first comment. We refer the reader to our response to that point for a more detailed discussion of cue validity and the distinction between exogenous and endogenous influences in our paradigm.

(4) The study would benefit from a clearer connection between the behavioral results and possible underlying neural mechanisms. How might the observed changes in contrast sensitivity relate to known physiological processes at the retinal, thalamic, or cortical level? The discussion could be strengthened by framing the findings within established models of attentional modulation or by referring to known effects of attention in the early visual cortex.

This is an important point, and we agree that framing the findings within established models of attentional modulation can strengthen the discussion. We believe that the normalization model of attention (Reynolds and Heeger, 2009; Herrmann et al., 2010) offers a useful framework for interpreting our behavioral findings, especially the attention-related changes in contrast sensitivity and asymptotic performance observed at the foveal scale. We have now added a more detailed discussion linking our results to this model and considering, explicitly as speculation, how known physiological processes at different stages may contribute to the observed effects in Discussion (lines 264-307).

(5) The ecological relevance of the results is not fully developed. The authors propose that the observed effects may resemble natural attentional shifts triggered by salient events, yet the brief, highly localized flashes used here are somewhat artificial. A more likely interpretation is that these mechanisms relate to oculomotor control within the fovea, perhaps reflecting preparatory activity for microsaccades or fine fixation adjustments. Considering this view could broaden the impact of the findings and link them to current discussions on the relationship between attention and oculomotor control.

We thank the reviewer for raising this important point regarding the ecological relevance of our findings, which we did not sufficiently address in the original manuscript. Although we briefly motivated scenarios that engage exogenous attention at high spatial resolution, such as detecting road signs or traffic lights at a distance while driving, we did not fully elaborate on how such attentional processes may link to downstream visual and oculomotor functions.

In our experiment, observers maintained fixation and avoided saccades throughout the trial. Nevertheless, in a subset of trials (on average 17% ± 3%), observers made saccades after stimuli disappeared and prior to providing a response. Typically, these movements were microsaccades with amplitudes smaller than 0.5°, directed toward the target location, in both valid and neutral trials. These saccades were discarded prior to the analyses performed in the manuscript. Inspired by the reviewer’s feedback, we decided to examine the saccade latency in these trials relative to the onset of the response cue to assess whether exogenous cueing influenced oculomotor timing. Notably, we observed an earlier onset of microsaccades in valid compared to neutral trials (71 ms ± 50 ms faster, P < 0.01). We have now added this observation as Figure 2 — Supplementary Figure 2 in the manuscript. Because the presence of an exogenous pre-cue was the only difference between the two trial types, the earlier microsaccade onset likely reflects exogenously triggered preparatory activity in the oculomotor system in response to the salient pre-cue. Such fine-grained attention may prime potential eye movements toward behaviorally relevant stimuli for further examination. This interpretation is consistent with the reviewer’s suggestion and supports a mechanistic link between exogenous attention and oculomotor behavior, extending the ecological relevance of our findings. This point has been added to the Discussion on lines 329 to 340.

We also conducted analysis to examine ocular drift behavior following the response cue. Although trials included in the manuscript analyses were constrained such that fixation during target presentation remained within a small window (10’ radius) around the fixation marker, we did not assess whether gaze subsequently drifted closer to the target location after the response cue. One possibility is that exogenous attention might bias ocular drift, shifting the preferred locus of fixation closer to the target. To address this, we computed the average Euclidean distance between gaze position and the target location following response cue onset for valid and neutral trials. However, we found no significant difference in gaze-target distance between valid and neutral trials (p = 0.57).

Although the spatial cueing approach has long been used to probe exogenous attention in a controlled manner in psychophysical experiments, we fully recognize the importance of understanding attention under more naturalistic viewing conditions that allow observers to freely move their eyes. Developing paradigms that incorporate more naturalistic, salient stimuli would be an important direction for future work, enabling investigation of exogenous attention in ecologically valid settings and its influence on sequential actions and processes, including oculomotor behavior.

(6) There is no statement about the availability of the data and code used for the experiment.

We have now added the data and code for the analysis pipeline to the Open Science Framework (OSF).

**Reviewer #2 (Recommendations for the authors):**

(1) The study could discuss the strength of the effect and how it relates to previous studies.

We thank the reviewer for raising this point. To facilitate direct comparison with the study by Jigo and Carrasco (2020), we computed attentional benefit as the ratio of contrast sensitivity between the valid and neutral conditions (now shown in Figure 3 — Supplementary Figure 4). In their data, the attentional benefit at 0° eccentricity peaked just below 4 CPD, with a ratio of approximately 1.2, corresponding to a ~20% increase in contrast sensitivity. This magnitude closely matches the benefit we observed for fine-grained attentional shifts within the foveola at spatial frequencies between 4 and 8 CPD (17% ± 12% and 16% ± 14% for 4 and 8 CPD, respectively). We have added this comparison to the Discussion (lines 246-262).

In addition, we acknowledge that prior studies have reported heterogeneous attentional effects, including pure contrast gain, pure response gain, or a mixture of the two. We now explicitly reference these findings in the Discussion and use the normalization model of attention (Reynolds and Heeger, 2009; Herrmann et al., 2010) to account for how differences in stimulus configuration, attention field size, and eccentricity may account for discrepancies between our findings and prior studies examining attention in the extrafovea or when broadly distributed across the fovea (lines 264-307).

(2) Minor details:

(a) The abstract mentions gaze-contingent-display, but if I understand correctly, the stimulus was not presented in a gaze-contingent manner.

That’s correct. Although stimuli were not presented gaze-contingently, we used a gaze-contingent calibration procedure (see Methods, lines 386-389) to achieve higher precision in localizing the line of sight. This increased accuracy was essential for selecting trials in which stimuli remained at the intended eccentricity relative to the preferred locus of fixation. To avoid potential confusion, however, we have removed this detail from the abstract.

(b) Line 361: What is the manual calibration the authors are referring to? It does not appear to be described.

The text has been updated to explain more explicitly what auto and manual calibrations are.

(c) Line 402: There may be a typo towards the end of the line "t0" should be "to"?

Text has been updated. Thank you.

(d) Line 405. What are the units of 30?

It’s in arcminutes. Text has been updated.

**Reviewer #3 (Recommendations for the authors):**

I found this paper very interesting, with a solid methodological approach and excellent data analyses. The authors present a well-designed psychophysical study that contributes valuable insights into the mechanisms of attention in the foveola. The methodology is rigorous, and the analyses are thoughtfully conducted and clearly presented.

That said, I would like to offer a few comments and suggestions for clarification and further consideration:

(1) Exogenous attention:

If a 100% spatially predictive cue is compared to a neutral cue, the observed attentional effect should not be described as (purely) exogenous, since the cue fully predicts where the post-cue will request a response. This situation represents a case in which attention is exogenously driven but endogenously maintained (see e.g., Chica et al., 2013, Behavioural Brain Research). I recommend clarifying this distinction in the manuscript (and title) to avoid conceptual ambiguity.

We thank the reviewer for raising this important conceptual point. We agree that because the pre-cue was 100% spatially predictive, the resulting attentional allocation cannot be considered purely exogenous. Although the abrupt, salient onset of the cue obligatorily triggers an exogenous shift of attention, its validity could also promote endogenous maintenance of attention at the cued location. Yet, several characteristics of our task strongly limit the extent to which such endogenous engagement could meaningfully influence performance. Endogenous attentional benefits typically emerge only after ~150-200 ms (Posner & Petersen, 1990; Carrasco, 2011), whereas our cue-target SOA was 100 ms, and the target remained visible for only 50 ms. Under these temporal constraints, any voluntary, slow endogenous enhancement would primarily occur after the stimulus offset. Thus, although endogenous maintenance is theoretically possible given the cue’s validity, it is unlikely to have substantially contributed to perceptual encoding in our task.

We also considered the possibility that our response cue (a retro-cue indicating the target location) might recruit endogenous attention to the internal perceptual representation. Importantly, however, this retro-cue was equally informative in valid and neutral conditions. Any enhancement driven by the retro-cue should therefore benefit both trial types to the same extent. The fact that we still observe a robust advantage in valid trials supports the conclusion that the performance improvements predominantly reflect fast, spatially specific exogenous facilitation rather than slower endogenous processes.

We have revised the manuscript to clarify that although the cue obligatorily triggers an exogenous attentional shift, its 100% validity could allow for endogenous attention maintenance as shown by Chica et al. (2013). We also added an explanation detailing why such endogenous contributions are unlikely to drive our main results, given the rapid cue-target timing in our task in Discussion (lines 319-327). Finally, to further prevent ambiguity, we updated the manuscript title to refer to “exogenously triggered attention,” rather than simply “exogenous attention.”

(2) Interpretation of statistical effects:

The statement "Therefore, asymptotic performance showed only independent, additive effects of frequency and attention, without a systematic influence of spatial frequency on the attentional benefit" seems not to be supported by the data, as the main effect of frequency was not significant.

We thank the reviewer for this helpful observation. We agree that the original phrasing did not accurately reflect the results, as the main effect of spatial frequency was not significant (p = .0545). We have revised the sentence to “Therefore, asymptotic performance reflected an effect of attention alone, with no detectable contribution of spatial frequency or of the interaction between spatial frequency and attention” to avoid implying such an effect (lines 210-211).

If data from two participants were missing in one condition, the authors should consider replacing this data with new participants.

We agree with the reviewer that having two observers with missing data in one condition is not ideal. However, the 20 cpd condition was deliberately positioned near the resolution limit at the tested eccentricity and was therefore extremely demanding. Observers also had to monitor two stimulus locations simultaneously, further increasing task difficulty. This condition was challenging for all observers and, despite testing up to the highest contrast, two of seven observers were unable to perform above chance, indicating that for a non-trivial fraction of observers, this condition was effectively unmeasurable with our paradigm. As noted in the manuscript, the 20 cpd condition also has a statistical limitation: thresholds clustered near the upper bound (approaching 100% contrast), compressing the dynamic range and markedly reducing variance relative to lower spatial frequencies, which violates the homoscedasticity assumption of linear models. For these reasons, we did not pursue additional data collection in this condition. Nevertheless, we report the data that were successfully obtained, as they remain informative about performance near the resolution limit.

We finally note that even when setting aside the 20 CPD condition, our data support this conclusion: comparisons between 4 and 12 CPD, as well as between 8 and 12 CPD, revealed large differences in the magnitude of the attentional benefit (d = 0.65, 95% CI [0.11, 1.18] and d = 0.62, 95% CI [0.08, 1.14], respectively). To further quantify these effects, we have added Cohen’s d to report the effect sizes for these spatial-frequency comparisons across texts in Results as well as in tables in Appendices.

(3) Sample size:

As this is a psychophysical experiment with many trials and few participants, I am curious about how the authors determined the appropriate sample size and the number of trials required to detect the expected effects. Given that many effects were found to be significant, it seems that statistical power was adequate; however, it would be helpful if the authors could explain how this issue was addressed a priori during experimental planning.

We appreciate that the reviewer raised this point. Please see the reply to the second point from Reviewer 1, who raised a related question about statistical power.

(4) Figure 2 clarification:

In Figure 2B, I do not fully understand the "Valid" and "Neutral" representation. Both conditions include a post-cue indicating the right position; however, in the neutral condition, there is a central fixation square, whereas in the valid condition, there is not. Please clarify this aspect of the figure. I think I understood the paradigm, but this part of the figure is misleading.

Precue only exists in valid condition. But there is a mistake where fixation marker is missing in valid condition in panel B.

We thank the reviewer for pointing this out. We have updated Figure 2 to explicitly show the sequence of valid vs. neutral trials. The fixation mark remained on the screen throughout the trial in both the valid and neutral conditions. After a 500 ms fixation period, an exogenous cue was presented for 30 ms in valid trials, followed by a 70 ms interval before stimulus onset. In neutral trials, no cue was presented, and the screen remained blank for 100 ms before the stimuli appeared. In conditions, a response cue would appear 50 ms after stimulus offset.
